# Supplementary material for: Human and Mouse Transcriptome Profiling Identifies Cross-Species Homology in Pulmonary and Lymph Node Mononuclear Phagocytes
Source: Cell Rep. Author manuscript; Available in PMC 2020 Nov 18. (PMC7673261; doi:10.1016/j.celrep.2020.108337)
Supplement: 1 [file NIHMS1643872-supplement-1.pdf]

Cell Reports, Volume *33*

## Supplemental Information

# Human and Mouse Transcriptome Profiling Identifies Cross-Species Homology in Pulmonary and Lymph Node Mononuclear Phagocytes

**Sonia M. Leach, Sophie L. Gibbings, Anita D. Tewari, Shaikh M. Atif, Brian Vestal, Thomas Danhorn, William J. Janssen, Tor D. Wager, and Claudia V. Jakubzick**

## Supplemental Information

Human

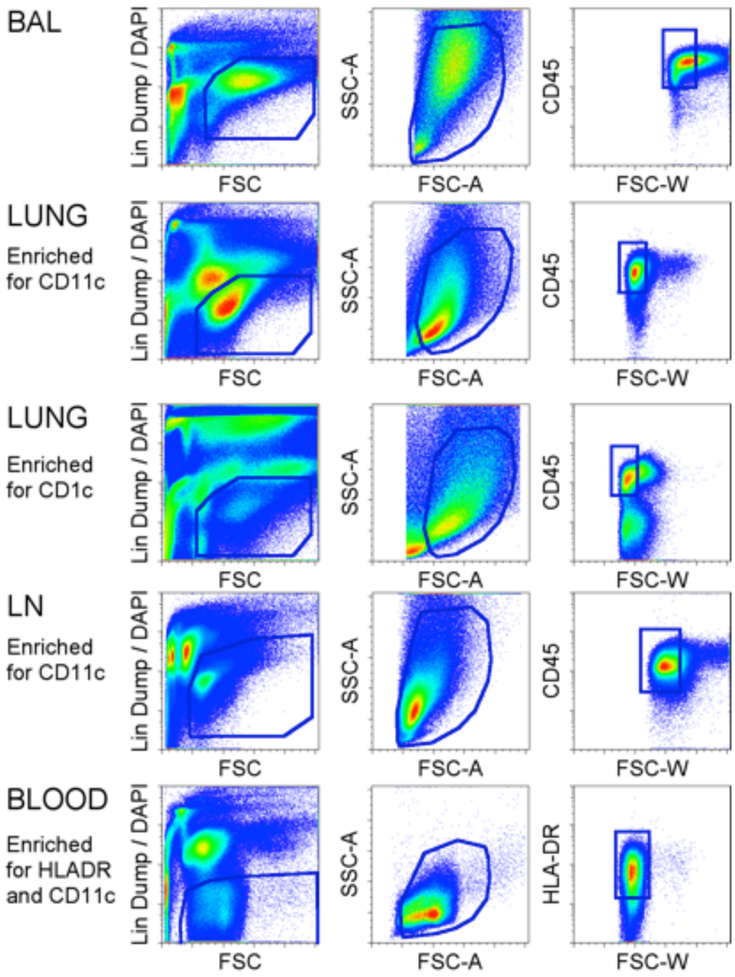


**Figure S1. Human MP initial sorting strategy,** Related to Figure 1.

Prior to identification of MPs, FACS gating was used to exclude Lin$^+$ cells, dead cells and doublets. Lineage (Lin) dump for each tissue included antibodies against CD3, CD20, CD15 and CD56. Dying cells were labeled with 4',6-diamidino-2-phenylindole (DAPI). Forward scatter (FSC) and side scatter (SSC) properties were used to distinguish whole cells from subcellular debris, and single cells from groups of two cells or more. Expression of CD45 was used to separate MPs from non-hematopoietic tissue cells.

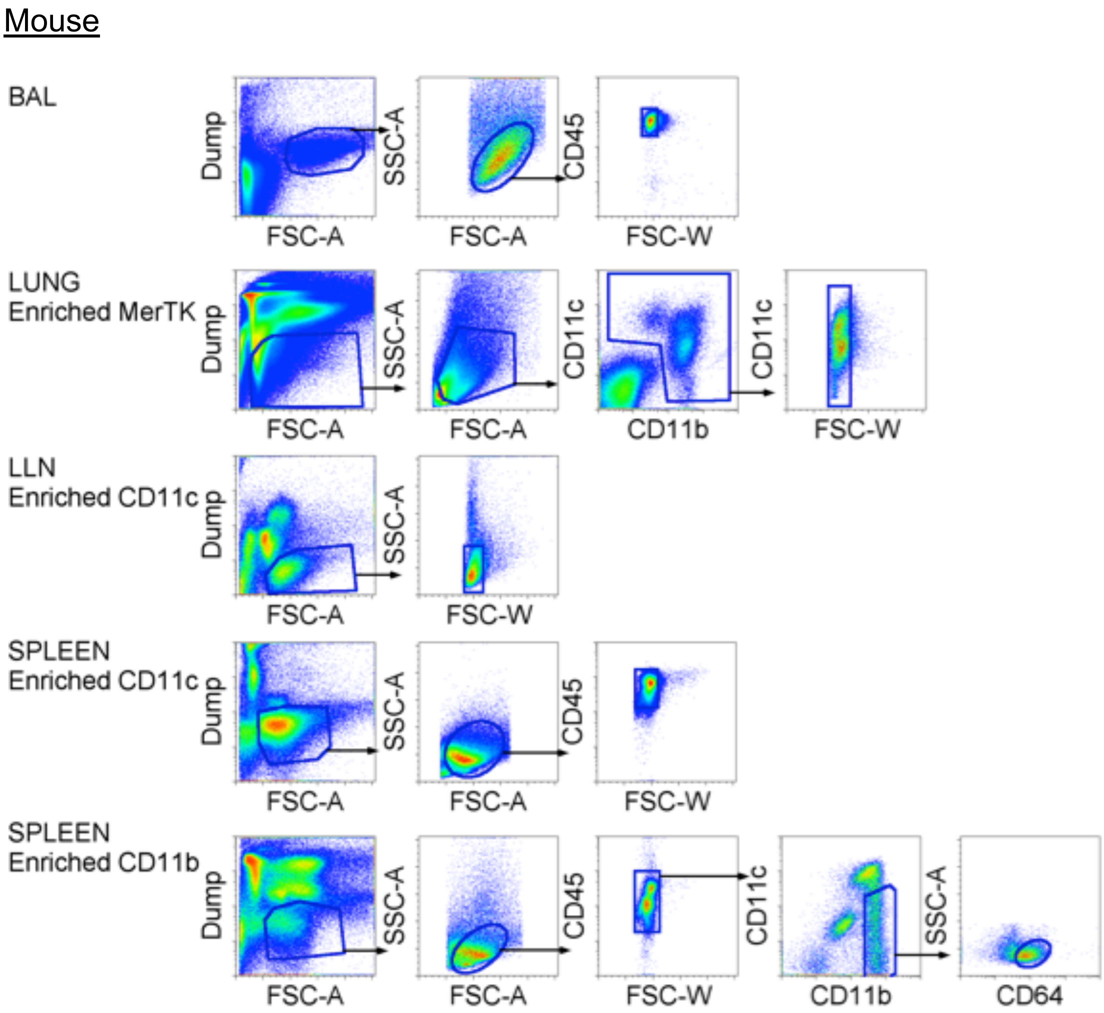


**Figure S2. Mouse MP initial sorting strategy,** Related to Figure 1.

Prior to identification of MPs, FACS gating was used to exclude Lin+ cells, dead cells, and doublets. Forward scatter (FSC) and side scatter (SSC) properties were used to distinguish whole cells from subcellular debris and single cells from doublets. Lineage (Lin) dump for each tissue included antibodies against CD3, B220, Ly6G and NK1.1. 4',6-diamidino-2-phenylindole (DAPI) was used to label dying cells. For DCs, tissue-specific dump antibodies included Siglec F, CD43 and Gr1 (Lung), Gr1 and CD64 ($CD11c^{+}$ LLN and Spleen). Additional gates confirmed hematopoietic ($CD45^{+}$) or myeloid lineage ($CD11c^{+}$ or $CD11b^{+}$).

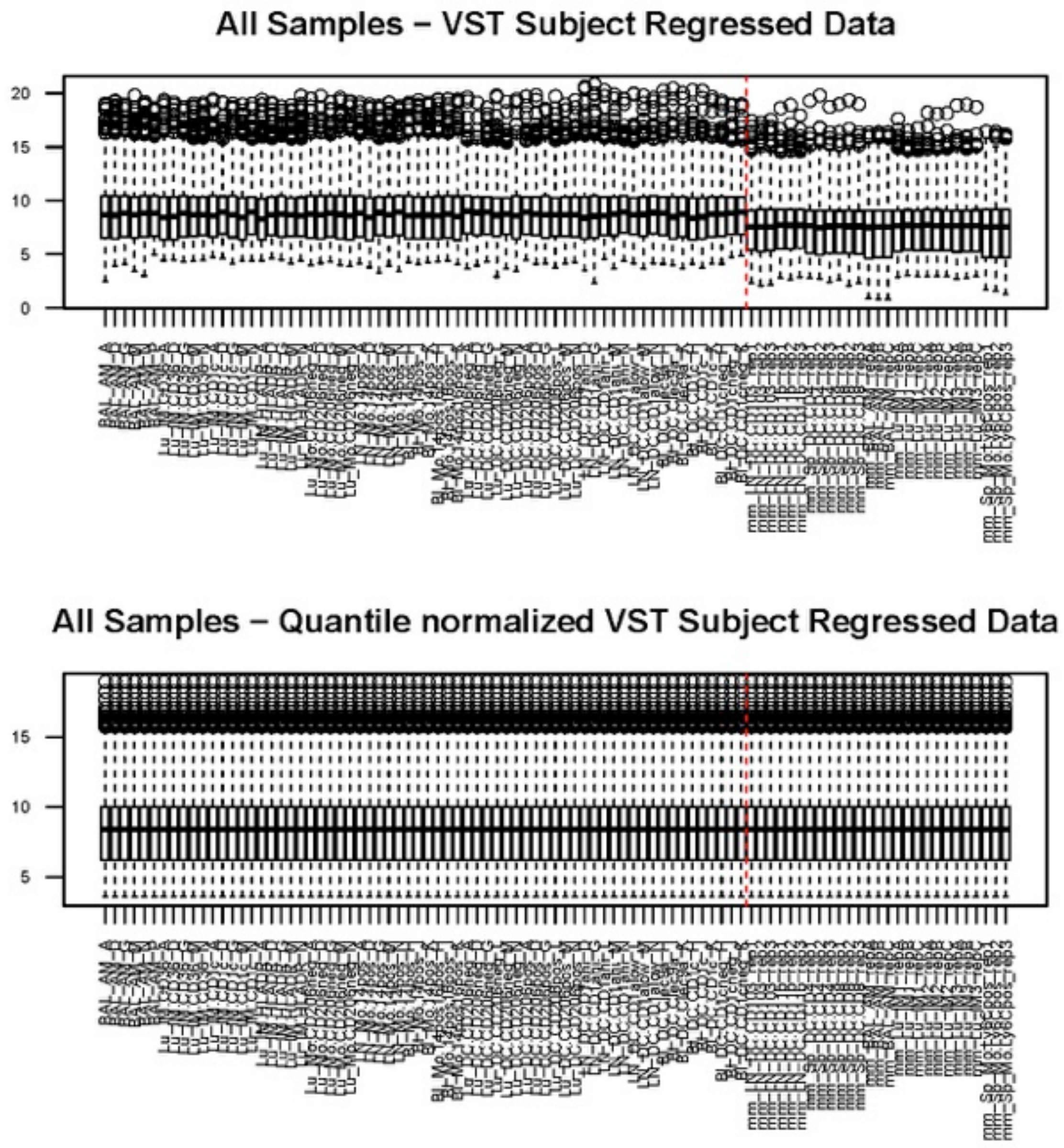


**Figure S3. Quantile normalization of VST subject-regressed data,** Related to Figure 3. Expression data is shown as boxplots for the VST subject-regressed data for all samples before and after quantile normalization. A vertical red line separates human subtypes on the left from mouse subtypes on the right. Note that before quantile normalization, the mouse samples generally had lower medians than the human samples.

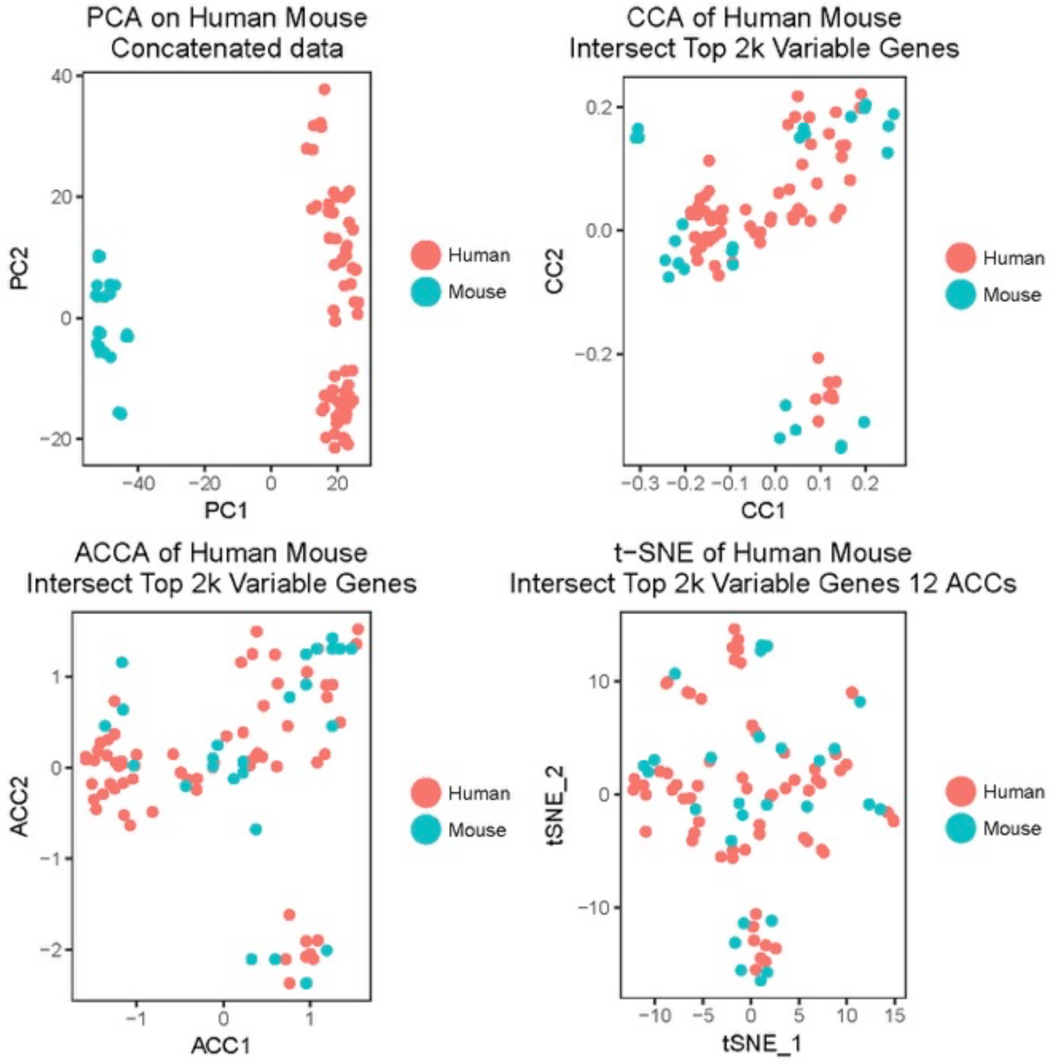


**Figure S4. Alignment of human and mouse cell types using Seurat v2.0,** Related to Figure 3. Human and mouse quantile normalized VST subject-regressed expression data is subjected to the alignment method in Seurat v2. A principal component analysis (PCA) of the combined human and mouse quantile normalized data shows clear separation per species even after quantile normalization. A canonical correlation analysis (CCA) of the genes in common among the top 2000 variable genes in each species is performed and visualized as the top 2 CCs for each species. The CCs are aligned via a dynamic time-warping algorithm to bring the two CC spaces into the same coordinate space. The top two resulting aligned CCs (ACCs) are used to visualize the combined data. T-distributed Stochastic Neighbor Embedding (t-SNE) of the top 12 ACCs shows that the human and mouse data no longer cluster solely by species.

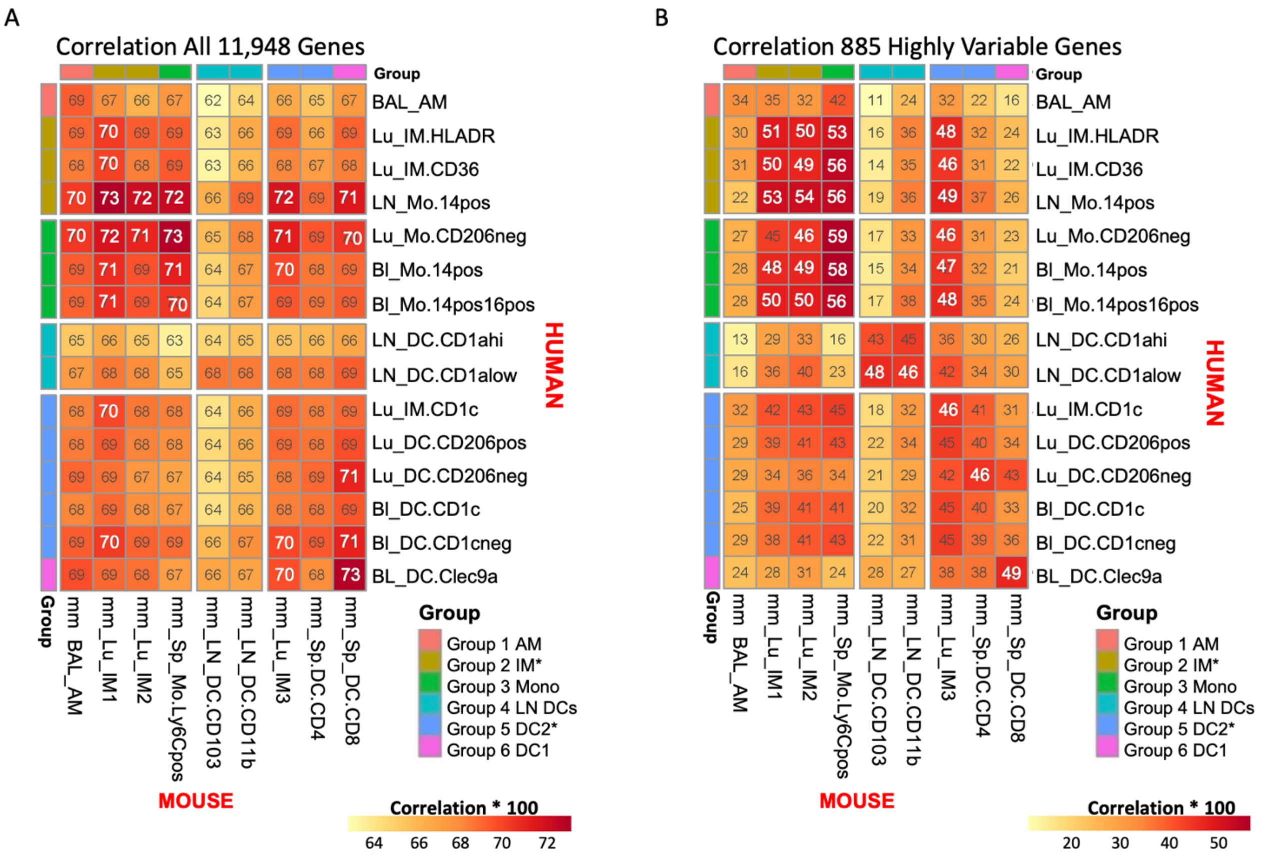


**Figure S5. Cross-species correlation of MP subtypes,** Related to Figure 4.

The median Pearson correlation is computed over all pairwise Pearson correlations between human samples for one cell type and mouse samples for one cell type. A) Correlation using all 11,948 autosomal homologous genes. Note that the correlation range is quite high and has a narrow range (r=64-72), such that any cell type appears equally closely related to most cross-species cell types. This may be attributed to a large fraction of all genes having low or constant expression. B) Correlation of the 885 most highly variable genes (hvg) shared among the top 2000 human hvgs and the top 2000 mouse hvgs. The range of correlations overall has dropped to r=20-50 but for a given cell type, multiple cross-species cell types seem nearly equally good homologs. Moreover, the best match suggested by A) does not always agree with the best match suggested by B), so determining best match by correlation is not robust to the input gene set.

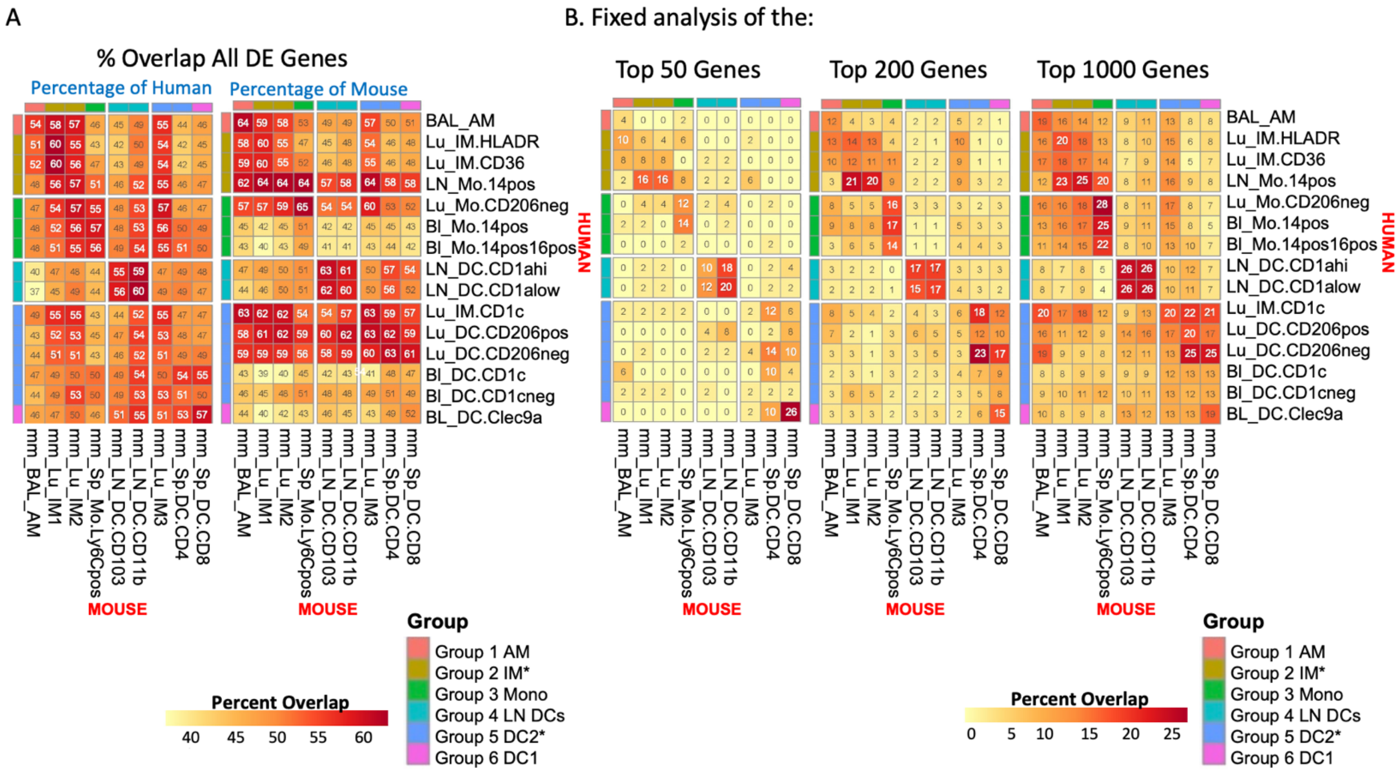


**Figure S6. Cross-species percentage overlap of marker gene sets,** Related to Figure 4.

A marker gene set includes genes with positive Z-score (see Methods) indicating over-expression in the cell type and p.adj > 0.05. Marker genes are ranked in decreasing order by score = [median scaled quantile normalized data]*Z-score. A) Percent overlap of all marker genes per cell type. Since the marker gene sets are not symmetric, values are shown using both human gene set sizes as the denominator for the overlap percentages and mouse. The number of marker genes ranges from approximately 4,000 to 7,000 of the original 11,948, so given the relative size of the marker gene set to the total gene set, high overlap is expected (40-68%). Note that using all DE genes is not discriminatively indicating which pairing is the best match. B) Percent overlap using fixed top marker gene sets. The percentage overlaps are symmetric given a fixed gene set size. The percent overlaps are smaller (0-25%) but are more discriminatory than using all DE genes. The maximum pairing changes given the size of the marker gene list, though several pairings are robustly suggested across all marker set sizes. This motivates the use of the average overlap percentage over the range 1-1000 as the criterion for selecting the best match.

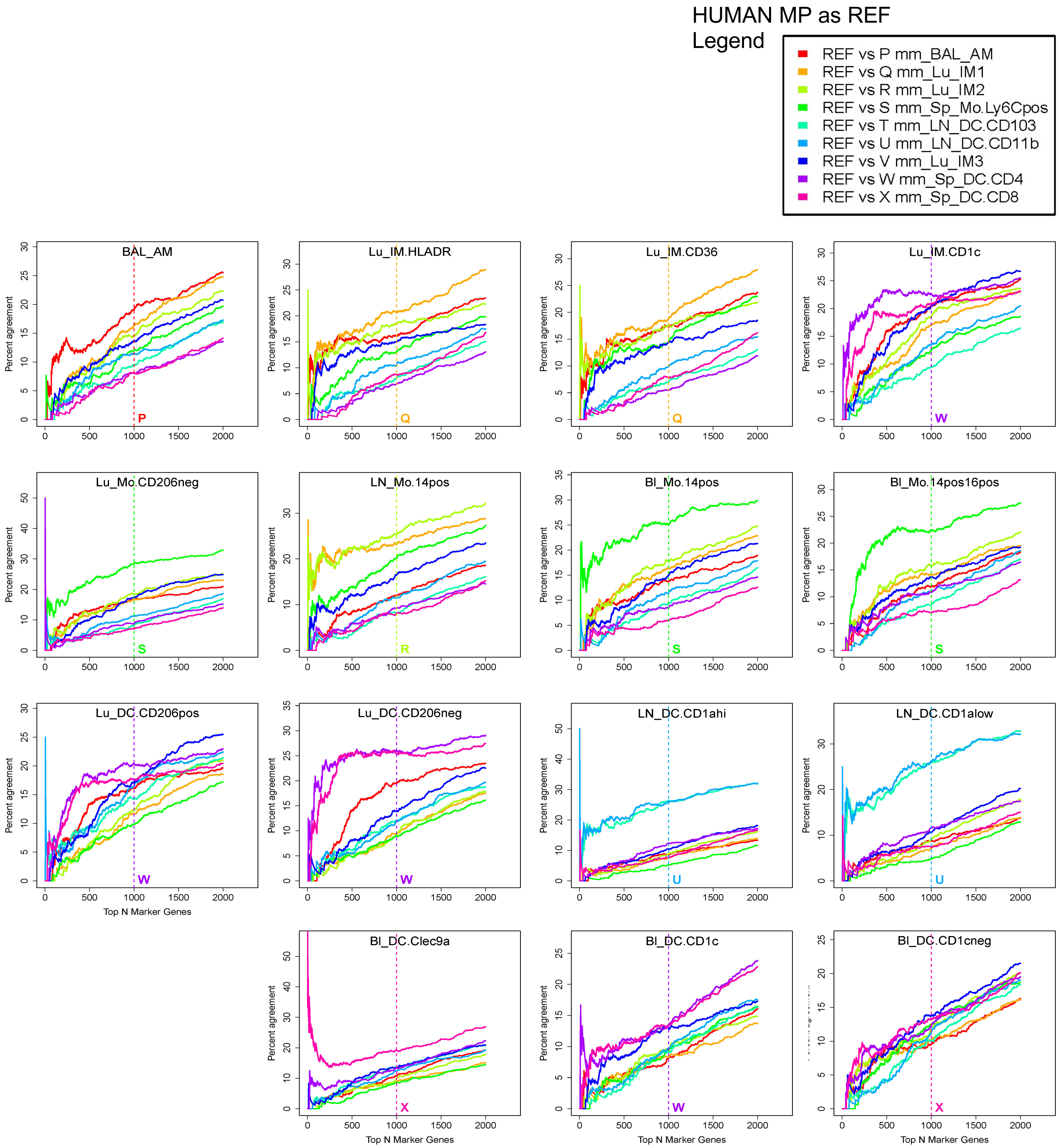


**Figure S7. Correspondence-at-the-Top (CAT) plots in human,** Related to Figure 4. Candidate marker genes are ranked by their score, which multiplies their expression level scaled relative to the median expression by their marker gene Z-score (see Methods). A) For each reference MP subtype, the proportion of agreement of ranked marker gene lists versus ranked marker gene lists for each candidate subtype in the other species is calculated for progressively larger list sizes and visualized with a Correspondence-at-the-Top (CAT) plot. Lines are labeled by letters from Figure 3 indicating the subtype compared to the reference shown in the title of the figure. A vertical line indicates which MP subtype has the highest mean CAT overlap across all list sizes from 1 to 1000 with respect to the reference.

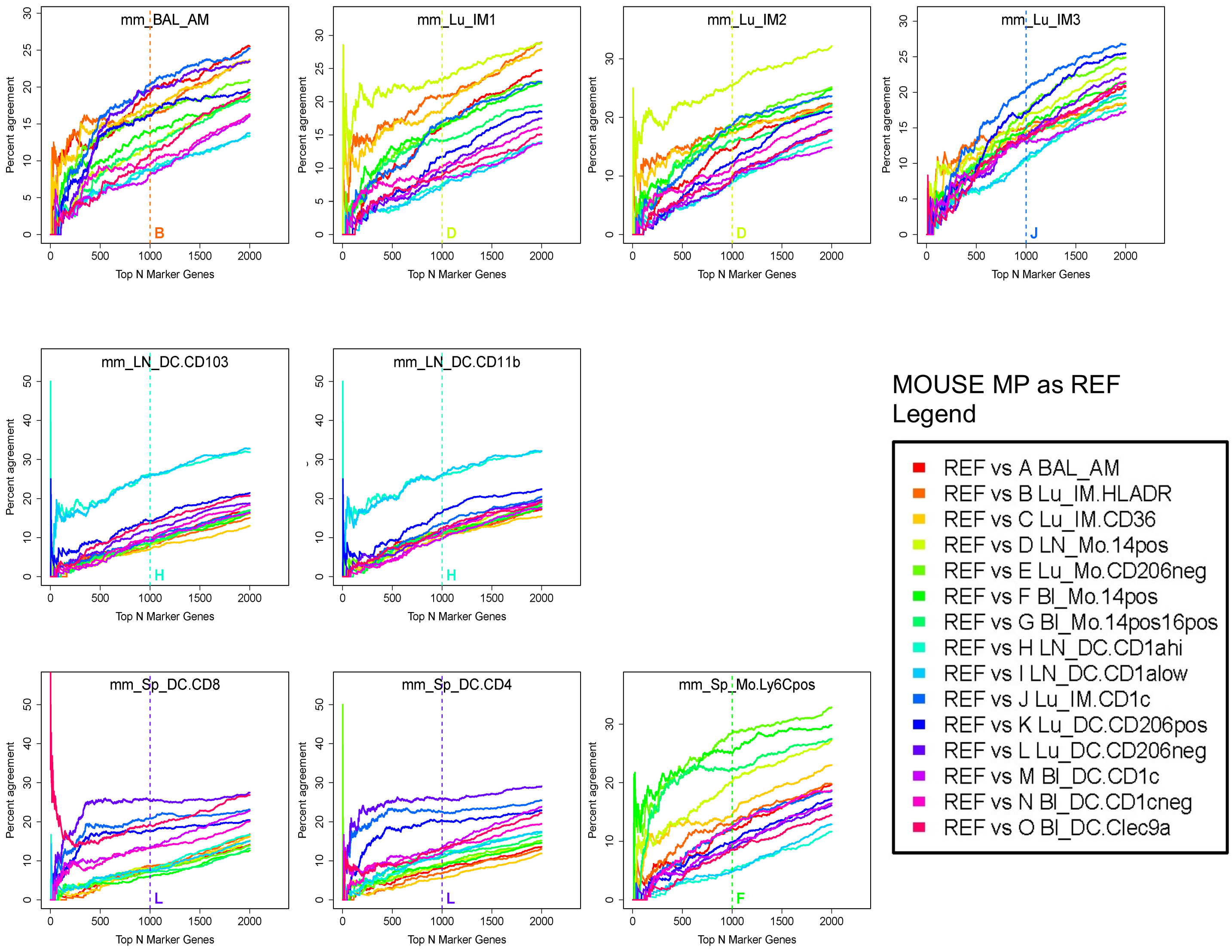


**Figure S8. Correspondence-at-the-Top (CAT) plots in mouse,** Related to Figure 4. Candidate marker genes are ranked by their score, which multiplies their expression levels scaled relative to the median expression by their marker gene Z-score (see Methods). A) For each reference MP subtype, the proportion of agreement of ranked marker gene lists versus ranked marker gene lists for each candidate subtype in the other species is calculated for progressively larger list sizes and visualized with a Correspondence-at-the-Top (CAT) plot. Lines are labeled by letters from Figure 3 indicating the subtype compared to the reference shown in the title of the figure. A vertical line indicates which MP subtype has the highest mean CAT overlap across all list sizes from 1 to 1000 with respect to the reference.

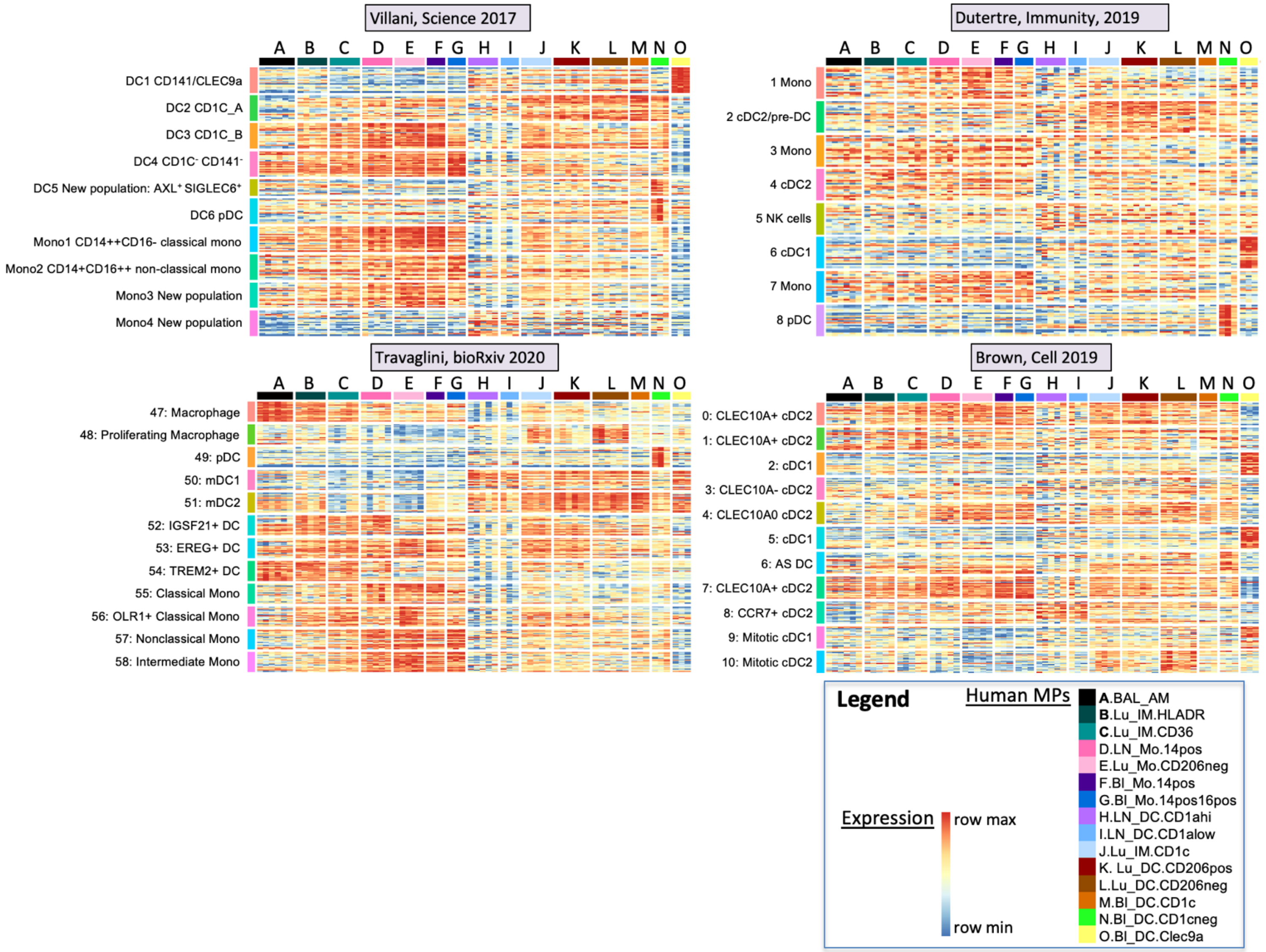


**Figure S9. Expression of previous DC and monocyte signature genes,** Related to Figures 5, 6, and 7.

Gene signatures from Villani et al., (Villani et al., 2017) Dutertre et al., (Dutertre et al., 2019) Brown et al., (Brown et al., 2019) and Travaglini et al., (Travaglini https://www.biorxiv.org/content/10.1101/742320v2] are shown as heatmaps of the top 20 signature genes expressed in the 15 human subtypes, denoted by the letters A through O. The data are scaled across the row by the gene min and max to emphasize in which subtype the gene is most expressed.

Correspondence of signature gene sets identified in these studies with the expected blood cell types in our MP data lends further validity to our investigation of the less well studied MP subtypes in lung and LN, as well as our cross-species MP analysis. As expected, our human blood Clec9a+ DCs corresponded most highly with the DC1 CD141/CLEC9a subset from Villani et al., (Villani et al., 2017) and the cDC1 subsets from

Dutertre et al., (Dutertre et al., 2019) and Brown et al., (Brown et al., 2019). The CD1c signatures from Villani et al., were expressed in our blood DC CD1c samples. Our blood DC CD1c- cells expressed genes from the pDC signatures in Villani and Dutertre et al., papers. The blood monocytes showed high expression of Villani's monocyte signatures: Blood CD14+ monocytes showed enrichment especially in Villani et al.'s Mono1 signature, while blood CD14+CD16+ monocytes showed enrichment in Villani et al.'s Mono2 signature. High expression of DC signature genes from Villani et al. and Brown et al., which show high expression in our monocytes, can be explained by the fact that those signatures were not derived by comparison to monocyte data. Dutertre et al. note that Villani et al.'s DC4 signature could correspond to CD16+ monocytes, which is also observed in our data. However, our LN DCs do not quite match any of the single-cell blood data sets, except perhaps Villani et al.'s Mono4 or Brown et al.'s CCR7+ cDC2 populations. The latter is interesting and ties back to the knowledge that human splenic DCs reflect a more mature signature than blood DCs, along with expressing CCR7.

**Table S1. EntrezGene ID and description of MP marker genes,** Related to Figures 5, 6 and 7.

| GENE NAME | EntrezGene ID | Description [Source:HGNC} |
|---|---|---|
| FABP4 | 2167 | fatty acid binding protein 4 |
| MARCO | 8685 | macrophage receptor with collagenous structure |
| PPARG | 5468 | peroxisome proliferator-activated receptor gamma |
| | | |
| S100A8 | 6279 | S100 calcium binding protein A8 |
| S100A9 | 6280 | S100 calcium binding protein A9 |
| CD14 | 929 | CD14 molecule |
| | | |
| XCR1 | 2829 | chemokine (C motif) receptor 1 |
| CADM1 | 23705 | cell adhesion molecule 1 |
| CLEC9A | 283420 | C-type lectin domain family 9, member A |
| WDFY4 | 57705 | WDFY family member 4 |
| | | |
| SERPING1 | 710 | serpin peptidase inhibitor, clade G member 1 |
| CES1 | 1066 | carboxylesterase 1 |
| MME | 4311 | membrane metallo-endopeptidase |
| IL17RB | 55540 | interleukin 17 receptor B |
| C1QA | 712 | complement component 1, q subcomponent, A chain |
| SPP1 | 6696 | secreted phosphoprotein 1 |
| MMP8 | 4317 | matrix metallopeptidase 8 |
| CARD11 | 84433 | caspase recruitment domain family, member 11 |
| | | |
| IDO1 | 3620 | indoleamine 2,3-dioxygenase 1 |
| MRC2 | 9902 | mannose receptor, C type 2 |
| CD8A | 925 | CD8a molecule |
| CD207 | 50489 | CD207 molecule, langerin |
| CCR7 | 1236 | chemokine (C-C motif) receptor 7 |
| | | |
| FOLR2 | 2350 | folate receptor 2 |
| SEPP1 | 6414 | selenoprotein P, plasma, 1 |
| LYVE1 | 10894 | lymphatic vessel endothelial hyaluronan receptor 1 |
| APOE | 348 | apolipoprotein E |
| CCL7 | 6354 | chemokine (C-C motif) ligand 7 |
| CCL2 | 6347 | chemokine (C-C motif) ligand 2 |
| | | |
| MMP9 | 4318 | matrix metallopeptidase 9 |
| F13A1 | 2162 | coagulation factor XIII, A1 polypeptide |
| MAFB | 9935 | v-maf avian musculoaponeurotic fibrosarcoma oncogene homolog B |
| MAF | 4094 | v-maf avian musculoaponeurotic fibrosarcoma oncogene homolog |
| TIMD4 | 91937 | T-cell immunoglobulin and mucin domain containing 4 |
| STAB1 | 23166 | stabilin 1 |
| | | |
| IL1R2 | 7850 | interleukin 1 receptor, type II |
| DUSP1 | 1843 | dual specificity phosphatase 1 |
| IL18R1 | 8809 | interleukin 18 receptor 1 |
| COL14A1 | 7373 | collagen, type XIV, alpha 1 |
| PDGFB | 5155 | platelet-derived growth factor beta polypeptide |
| | | |
| CCL22 | 6367 | chemokine (C-C motif) ligand 22 |
| CCL17 | 6361 | chemokine (C-C motif) ligand 17 |
| LAMP3 | 27074 | lysosomal-associated membrane protein 3 |

**Table S2. Demographics for RNA-seq,** Related to Figure 3.

| Age (years) | Smoking history | Sex | Tissue contributed |
|---|---|---|---|
| 40 | light/ex smoker | M | BAL, Lung and LLN |
| 23 | light smoker | M | BAL, Lung and LLN |
| 13 | 4 week smoker | M | BAL, Lung and LLN |
| 1.4 | non smoker | M | BAL, Lung and LLN |
| 77 | non smoker | F | BAL, Lung and LLN |
| 62 | ex smoker | F | BAL, Lung and LLN |
| 35 | non smoker | F | BAL, Lung and LLN |
| 39 | non smoker | M | BAL only |
| 31 | non smoker | F | Blood |
| 30 | non smoker | F | Blood |
| 24 | non smoker | F | Blood |
